# Targeting subchondral bone in osteoporotic osteoarthritis

**DOI:** 10.1186/s13075-014-0494-0

**Published:** 2014-11-25

**Authors:** Jorge A Roman-Blas, Gabriel Herrero-Beaumont

**Affiliations:** Bone and Joint Research Unit, Service of Rheumatology, IIS Fundación Jiménez Díaz, Universidad Autónoma, Av. Reyes Católicos 2, 28040 Madrid, Spain

## Abstract

The identification of well-defined phenotypes along the course of the disease may open new avenues for personalized management in osteoarthritis (OA). *In vivo* research carried out in various animal models as well as epidemiological and clinical data support the existence of a particular phenotype – osteoporotic OA. In fact, subchondral bone has become a potential therapeutic target in OA. Depending on the ratio between formation and resorption, subchondral bone remodeling can culminate in either a sclerotic or an osteoporotic phenotype. Patients with osteoporotic OA may thus achieve clinical and structural benefit from treatment with bone-targeted interventions.

Subchondral bone has become a potential therapeutic target in osteoarthritis (OA). In a previous issue of *Arthritis Research & Therapy*, Wang and colleagues demonstrate that osteoporosis aggravates cartilage damage in an experimental model of knee OA in rats [1]. Interestingly, the authors also describe that extracorporeal shockwave therapy (ESWT), a mechanical therapeutic intervention probably acting at subchondral bone, may reduce OA progression [1]. The significance of these findings in experimental osteoporotic OA relates to the search for well-defined phenotypes in human OA that will lead to personalized therapy.

The controversy regarding the relationship between subchondral bone quality and cartilage integrity originates from the complex biological and mechanical nature of the osteochondral junction [2]. OA progression is often accompanied by increased subchondral bone remodeling that enables mechanical forces to dynamically modify its structure. Depending on the ratio between formation and resorption, subchondral bone can exhibit either a sclerotic or an osteoporotic phenotype [3]. These phenotypes may represent up to 70% and 30% of patients in daily practice, respectively [4]. Furthermore, OA in females can display a different pathogenic profile from OA in males. In this sense, it is reasonable to underline the consequences of estrogen deficiency during menopause [5]. A low estrogen state could induce a deleterious effect on all articular tissues of the knee joint, the subchondral bone being particularly affected due to its capacity for high bone turnover. Thus, during early post menopause, estrogen deficiency may be a risk factor for the development of knee OA. Taking all these facts into consideration, the characterization of patients with either sclerotic or osteoporotic OA phenotypes may enable individualized targeted therapy [3].

The effects of estrogen deficiency on the knee joint have been reported in various experimental animal models of OA. The findings obtained by Wang and colleagues on subchondral bone quality and articular cartilage damage support previous research carried out in rabbits, in which osteoporosis aggravated instability-induced OA [6]. In this combined model, the induction of systemic and subchondral osteoporosis associated with increased bone remodeling resulted in worse cartilage damage compared with control animals. Greater fragility of the subchondral bone was suggested to account for the aggravation of cartilage damage when early OA and osteoporosis coexist [7]. In a further study carried out in the same model, the intermittent administration of parathyroid hormone 1-34, a bone-forming agent, was used to increase subchondral bone density and quality [8]. As a consequence, the improvement of subchondral bone integrity was associated with reduced progression of cartilage damage in OA preceded by osteoporosis. In a similar approach, the inhibition of bone resorption by pamidronate in osteoporotic mice alleviated the instability-induced OA histological score with a reduction in the expression of aggrecanases [9]. Several experimental models therefore indicate that osteopenia/osteoporosis induces an accelerated progression of knee OA that can be reversed not only by bone-forming agents but also by antiresorptive drugs.

These findings in animal models could be translated to humans, and together with epidemiological and clinical data they support the existence of a particular phenotype – osteoporotic OA [10]. Indeed, this phenotype characterized by decreased density and high remodeling at subchondral bone defines a subgroup of patients treatable with specific agents. In fact, beneficial effects of bone-acting drugs in OA are increasingly reported, but reliable conclusions regarding their efficacy are hindered by methodological drawbacks in study design [10]. Identifying patients with osteoporotic OA may improve the success of bone-directed agents.

The original approach of using ESWT in OA by Wang and colleagues remains intriguing. These authors have reported previously that the application of ESWT to subchondral bone of the proximal tibia showed a chondroprotective effect in the initiation of knee OA and regression of established OA of the knee in rats. These effects were attributed to the ESWT multifunctional actions on cartilage and bone. Yet achieving such beneficial effects in this osteoporotic OA model suggests that the main mechanism of action of ESWT may be improving subchondral bone structure [1]. However, some limitations on the study design and the lack of adequate standardization of dosages and optimal frequency, as well as little information regarding the molecular mechanisms underlying the effects of ESWT, hold back the achievement of solid results. In any case, this study points out the potential benefit of nonpharmacological interventions aiming to improve mechanical properties of articular tissues in OA.

In summary, the study by Wang and colleagues further supports the existence of the osteoporotic OA subtype and the potential benefit of bone-acting therapeutic interventions. Consequently, the identification of patient phenotypes along with the discovery of specific therapeutic interventions targeting relevant pathogenic mechanisms during the course of the disease could lead to a personalized approach to the management of OA.

## Abbreviations

ESWT: Extracorporeal shockwave therapy
OA: Osteoarthritis
